# On the reconceptualization of Alzheimer’s disease

**DOI:** 10.1111/bioe.12516

**Published:** 2018-10-10

**Authors:** Maartje H. N. Schermer, Edo Richard

**Affiliations:** ^1^ Department of Medical Ethics and Philosophy of Medicine University Medical Center Rotterdam The Netherlands; ^2^ Department of Neurology Radboud University Medical Center Nijmegen The Netherlands

**Keywords:** Alzheimer’s disease, biomarkers, dementia, disease concepts, early diagnosis, pragmatism

## Abstract

In the hope of future treatments to prevent or slow down the disease, there is a strong movement towards an ever‐earlier detection of Alzheimer’s disease (AD). In conjunction with scientific developments, this has prompted a reconceptualization of AD, as a slowly progressive pathological process with a long asymptomatic phase. New concepts such as ‘preclinical’ and ‘prodromal’ AD have been introduced, raising a number of conceptual and ethical questions. We evaluate whether these new concepts are theoretically defensible, in light of theories of health and disease, and whether they should be understood as disease or as an at‐risk state. We introduce a pragmatic view on disease concepts and argue that an evaluation of the reconceptualization of AD should also take its aims and effects into account, and assess their ethical acceptability. The reconceptualization of AD is useful to coordinate research into preventive strategies, and may potentially benefit future patients. However, in the short term, early detection and labelling of ‘preclinical AD’ can potentially harm people. Since there is no treatment available and the predictive value is unclear, it may only create a group of ‘patients‐in‐waiting’ who may suffer from anxiety, uncertainty and stigmatization, but will never actually develop dementia. We conclude that only if the promise of preventive medication materializes, will the reconceptualization of AD turn out unequivocally to be for the better. Otherwise, the reconceptualization may do more harm than good.

**The case**

A 75‐year‐old man visits a memory clinic because of memory complaints over the past 6 months. His wife was the first to notice. He used to have a fantastic memory, but now he sometimes forgets conversations they had. He also has a tendency to repeat a story he has told not so long ago. The couple live independently and have an active social life. The ‘patient’ plays tennis in a near‐by village, where he travels to by car or bike, depending on the weather. They have always done the financial administration of their household together. This has not really changed, although he has become slightly insecure and lets his wife take more decisions than he used to. On formal cognitive testing he has slight memory impairment, but no impairment in other cognitive domains such as executive functioning, language or visuospatial functioning.

This person clearly does not have dementia. In spite of some memory impairment he has almost normal and independent daily functioning. But the question is whether he is showing signs of a very early stage of a devastating brain disease that will eventually lead to dementia. Should we aim to label this person as having a disease at this point? And can we actually predict with sufficient accuracy if and when this person will eventually develop dementia? And if so, would this person benefit from this prediction?

## 1 INTRODUCTION

In the hope of future treatments to prevent or slow down the disease, there is a strong movement towards an ever‐earlier detection of Alzheimer’s disease (AD). It is believed that brain changes presumed eventually to lead to dementia start to develop years to decades before clinical symptoms of dementia occur.^1^ It seems attractive to develop treatments that can stop or slow down these changes as early as possible, even before symptoms of cognitive impairment arise. It is now possible to detect certain biomarkers – proteins in cerebrospinal fluid (CSF) and protein depositions on neuroimaging – that are presumed to reflect the early brain changes that may eventually lead to clinical dementia, in persons with no or only mild cognitive impairment. Biomarkers are thus increasingly seen as means for early detection of the disease.^2^

In line with these scientific developments, a reconceptualization of the notion of AD is taking place. Instead of being defined by the clinical syndrome of dementia, AD is more and more depicted as a well‐defined slowly progressive pathological process with a long asymptomatic phase. A new lexicon has even been proposed and new concepts and definitions have been introduced, such as ‘preclinical’ and ‘prodromal’ AD in those with no or only mild symptoms.^3^ While currently mainly used in research, these new concepts of preclinical and prodromal AD, as well as the use of biomarkers that define these ‘conditions’, which were originally intended for research purposes, are now gradually entering clinical practice.

This reconceptualization of AD, and the concomitant emerging use of biomarkers aiming to detect biological evidence of a presumed pathological process, raise a number of conceptual and ethical questions. First, from a theoretical perspective it is questionable whether a state with abnormal biomarkers but without overt clinical symptoms should be considered as a disease, or rather as an at‐risk state. For the persons it concerns, the distinction between risk prediction and a very early diagnosis of a much‐feared disease may not be that clear. This is further complicated by the fact that it is still contested how accurately biomarkers can predict future symptomatic disease. Hence, the exact meanings of biomarker‐based categorizations of preclinical and prodromal AD are unclear.

Second, from a moral perspective, the unclear predictive abilities of current biomarker tests and the current lack of meaningful treatment and prevention options – a lack of actionability – make it questionable whether the categorization of people as having an asymptomatic early phase of AD is defensible. It could create a group of ‘patients in waiting’ who may suffer from anxiety and fear for the future, but may never actually develop dementia.

Third, the motives for aiming at early detection are not always clear. Of course there is the intrinsic motivation of clinicians and researchers alike to stop the progression or even prevent the onset of dementia. Another driving factor is constant technological advancement, leading to the ability to detect molecular disease markers in body fluids as well as on neuroimaging. This technological advancement brings inevitable financial incentives, both for those who develop the biomarker tests (‘diagnostic tests’) and for those who foresee a large target population of people with an early stage of a disease for their new drugs. The persons it concerns are often driven by fear or the hope that something may be done to prevent a feared future with dementia; this may render them vulnerable to misconceptions and unrealistic expectations.

In this article we aim to address these important conceptual and ethical questions that result from the recent reconceptualization of AD. We will discuss whether the new concepts of preclinical and prodromal AD are theoretically defensible and ethically desirable and consider their implications for medical practice.

## 2 FROM ALOIS ALZHEIMER TO CURRENT DISEASE CRITERIA

Historically, the notion of AD has changed considerably since Alois Alzheimer first described the case of Auguste D who developed ‘pre‐senile dementia’ in her early fifties, in 1907. For decades, AD was considered a rare form of dementia with an onset at relatively young age. This as opposed to the common ‘senile dementia’, which was considered to be attributable to cerebral atherosclerosis. It was not until the 1970s that neurologist Robert Katzman first suggested that most dementia cases, also in later life, should be considered as AD.^4^ This led to a radical change in disease concepts and a dramatic increase in interest of the scientific world in dementia. In the 1980s the proteins of which the plaques and tangles Alois Alzheimer described consisted were identified as amyloid‐β and tau, respectively. This further enhanced research and fueled the hope for earlier diagnosis and potential treatment.

In parallel, clinical concepts started shifting, and the early disease stages, before the onset of dementia – which is defined by impairment in daily functioning – got more attention. In the late 1990s, ‘mild cognitive impairment’ (MCI) was introduced as an entity defined by cognitive impairment, but not severe enough to impair daily functioning.^5^ It became technically possible to assess the presence of amyloid‐β and tau protein in CSF and even using nuclear imaging of the brain. Combining early clinical detection with detecting these biomarkers has triggered a completely new way of thinking about the disease, shifting from a clinical diagnosis based on functioning to a biological diagnosis with or without cognitive dysfunction.

From 2009 onward, this has led to the development of two new conceptual frameworks, by the U.S. National Institute on Aging and the Alzheimer’s Association (NIA‐AA) and an international working group for new research criteria for the diagnosis of AD (IWG).^6^ According to these frameworks, AD is defined by a pathological process characterized by a specific sequence of brain changes, which develop over a long period of time and may or may not become symptomatic during life. Resulting from these frameworks, a new lexicon and new criteria have been proposed to capture these changing conceptualizations. Biomarkers, which can be detected using CSF analysis, magnetic resonance imaging (MRI) scanning and positron emission tomography (PET) scanning, play an important role in these definitions (Table 1). The main difference between the two conceptual frameworks is that according to the NIA‐AA criteria one can have AD in the absence of any symptoms, whereas, according to the IWG, people without symptoms can only be labelled as ‘at risk for AD’. Both frameworks consider people with MCI and abnormal biomarkers as having AD, irrespective of whether their cognitive impairment is progressive and will lead to dementia.

**Table 1:** Simplified representation of the new nomenclature for AD as suggested by the two working groups

State	Biomarker presence	NIA‐AA disease definition	IWG disease definition
No cognitive impairment	+	Preclinical AD stage 1–2	Asymptomatic at risk for AD
Subtle cognitive impairment	+	Preclinical AD stage 3	
MCI	+	MCI due to AD	Prodromal AD
	−	MCI unlikely due to AD	MCI
Dementia	+	Probable AD dementia	AD dementia
	−	Dementia unlikely due to AD	Dementia, not due to AD

Note

AD = Alzheimer’s disease; MCI = mild cognitive impairment.

So over the last century AD seems to have changed from a rare presenile form of dementia, clinically defined and characterized by the presence of cognitive impairment leading to functional decline, of which a certain diagnosis could only be made post‐mortem, to a common biologically defined condition in older people with or without cognitive impairment. The current views as represented in Table 1 illustrate the conception of AD as a slowly progressive pathophysiological process that will eventually lead to symptoms that will worsen and ultimately result in full‐blown dementia. This process can presumably be detected early on (before any clinical symptoms are present) and is – also presumably – unidirectional. The often‐invoked metaphor of a cascade (as in the amyloid cascade hypothesis^7^) indicates necessary progression in a single direction: inevitable decline.

The current amyloid cascade model is, however, hypothetical. The biological mechanisms are still insufficiently understood and it is not certain that the brain changes that define the condition do inevitably lead to symptoms. It is known that a considerable proportion of people dying in old age without cognitive impairment have a substantial load of amyloid‐β and abnormal tau protein, considered to be the hallmarks of AD, in their brains.^8^ In contrast, these changes are not always found in those dying at a greater age with clear symptoms of dementia, which was clinically diagnosed as Alzheimer’s dementia.^9^ Moreover, of those with MCI, only 5–15% develop dementia per year, depending on the population under study. Those with MCI who have these ‘AD biomarkers’ in their brain, do not always develop further cognitive decline, and after several years a considerable proportion has not developed dementia.^10^ For people without cognitive impairment, data are scarce, but do suggest that abnormal biomarkers are common and do not indicate imminent dementia in the majority of people.^11^

This brings us back to our main question: how should we evaluate the reconceptualization of AD? Are the new disease concepts theoretically defensible? And are they ethically desirable? We will start with the first question – which will eventually lead us to the second.

## 3 ALZHEIMER’S DISEASE AND THEORIES OF HEALTH AND DISEASE

In order to consider the defensibility of the new disease concepts proposed in the AD field, we take a look at how health and disease in general have been conceptualized in the philosophy of medicine literature. Two well‐known and influential theories in this field are those of Christopher Boorse and Lennart Nordenfelt. There is a long‐standing debate between proponents of these two theories, mainly focusing on the question of whether health and disease are value‐free concepts. Boorse and other naturalists claim that they are, while adherents of normativist theories such as Nordenfelt’s claim they are not.^12^ This difference in approach becomes apparent in the fact that Boorse’s biostatistical theory (BST) considers pathophysiological processes to be the objective, natural essence of disease, whereas Nordenfelt’s holistic theory of health (HTH) considers the ability to attain vital goals, and hence the impact of clinical symptoms on the lives of people, to be the fundamental aspect. This is also important for the discussion about AD.

Boorse’s BST defines health as statistically normal biological functioning of all parts of the organism, contributing to survival and procreation.^13^ He defines disease as any disturbance of health, and thus as statistically abnormal functioning of one or more parts of the organism. According to this theory, whether ‘preclinical AD’ is a disease depends on the question whether there actually is a pathological process going on in the brain.^14^ Boorse would say that if the nerve cells, neural networks, or brain tissues involved function with less than typical efficiency, this implies that a disease process is present, even if there are no clinical symptoms. This is in line with the proposal of the NIA‐AA to consider the asymptomatic stages of AD as disease states because, according to their view, pathophysiological changes are present in the brain, and these are seen as the essence of the disease. An interesting problem here is that with increasing age, amyloid‐β and abnormal tau are present in an increasing number of people with increasing severity, eventually in up to over half of those over 80, irrespective of cognitive functioning.^15^ So, if we follow Boorse’s understanding of normal functioning as statistically normal *in a specific reference group *the presence of amyloid‐β and high abnormal tau levels do not constitute disease but are normal in this age group.^16^

Nordenfelt’s HTH, on the other hand defines health as being in a bodily and mental state that is such that one has the ability to realize all one’s vital goals.^17^ A disease is a bodily state or process that tends to reduce health, and so tends to prevent people from realizing their vital goals. According to this view, at first glance, ‘preclinical AD’ is not a disease. Since there are no symptoms, the ability to attain vital goals is not compromised, and hence the person in question can be considered healthy. Several medical commentators have also stressed that, in the absence of symptoms, preclinical AD should not be considered a disease.^18^ This view is in line with the proposal of the IWG, which states we should speak of disease only when symptoms are present, i.e., in the prodromal and dementia stages. Asymptomatic presence of AD pathology, in their view, does not constitute disease but rather a risk factor for disease (‘asymptomatic at risk for AD’ as they call this ‘condition’). Even in prodromal AD, when there are only limited symptoms and it is uncertain if and when this will lead to dysfunction in daily life, it is questionable if, according to the HTH, these people should be considered as having a disease.

However, it is not uncommon in medicine to recognize asymptomatic disease – for example asymptomatic cancer, or asymptomatic renal failure – and Nordenfelt would probably agree that these are diseases because over time they do indeed tend to reduce the ability to attain vital goals. The bigger the chance that a pathological process will lead to symptoms within a short timeframe, the more reason there is to call this condition a disease, according to the HTH. With regard to asymptomatic presence of AD biomarkers, however, it is as yet unclear how high the chances of developing symptoms are, while it *is* clear that not all biomarker‐positive individuals will develop cognitive decline. Therefore we believe that, in line with the HTH and given the present state of scientific evidence, prodromal AD should not be called a disease.

In summary, at present the theoretical defensibility of preclinical AD as being an asymptomatic disease state, as proposed by the NIA‐AA and in line with Boorse’s theory of disease, is doubtful because scientific evidence is insufficient and because it is unclear whether at a certain age it may be a sign of normal aging. The conceptualization of the IWG, that understands the asymptomatic presence of biomarkers (‘preclinical AD’ according to NIA‐AA) to be a risk factor for developing symptomatic disease is more in line with Nordenfelt’s theory that sets the impact of disease symptoms on a person’s life – sometimes also referred to as ‘illness’^19^ – centre stage. This usage of the term ‘disease’ as referring to *symptomatic* disease is more in line with that of ordinary people and clinical practitioners, whereas the NIA terminology, like Boorse’s theory, reflects the pathologist’s and researcher’s perspective.

## 4 A PRAGMATIC APPROACH TO DISEASE CONCEPTS

The debate about definitions of disease in the philosophy of medicine has been focused primarily on the dispute between naturalist and normativist positions, and about what constitutes the essence of health and disease. We agree, however, with an argument by Kingma^20^ that a social–constructivist approach to health and disease may be more fruitful, because it can unite those traditional positions and move the debate forward. Moreover, and very important for our present evaluation of the reconceptualization of AD, social–constructivism does not merely focus on health and disease as general concepts, but recognizes that defining and demarcating disease is a human activity; it is always a matter of discussion, negotiation, consensus‐seeking and agreement among the experts involved. The processes of redefining AD by the working groups mentioned above poses an excellent illustration of such a process.^21^

Social–constructivists do not attempt to give abstract definitions of what health and disease *really are*, but claim that this is contingent on human decisions and agreements.^22^ The entities we distinguish and the demarcation lines we draw are the result of human activity, not simply given by nature. Another way to put this is to say that diseases are not ‘natural kinds’ but ‘practical kinds’, and can change over time.^23^ The mere fact that two different groups of experts come up with similar, yet not identical proposals of how to ‘carve up’ the landscape of AD, illustrates this point. Scientific work as performed in these groups consists of drawing up definitions, agreeing on classifications, and providing argumentative and empirical justification for the choices made in this process. Social–constructivism claims that this is not a matter of ‘discovering’ some underlying true structure of the world, but rather of constructing useful and justifiable concepts and entities. Interestingly, this constructive nature of the reconceptualization of AD appears to be recognized by the medical research community itself: an editorial commentary in the Lancet speaks of ‘organising the language of AD’;^24^ another talks of preclinical and prodromal AD as ‘conceptual constructs’.^25^ Moreover, it has been argued that various interests, beyond the interest of the ‘patient’, e.g., of pharmaceutical companies and of researchers, are at stake in this (re‐)construction of AD.^26^

A shortcoming of a purely social–constructivist approach, however, is that it is merely descriptive and not normative. It gives an account of how concepts of disease and disease classification are developed. As such, it offers no criteria to decide whether a certain conceptualization is a good one, or to determine whether one conceptualization, definition, or classification is better or worse than another. We therefore propose a pragmatic approach to concepts and classifications of disease.^27^ Philosophical pragmatism understands definitions, concepts and classifications as *tools*. From this perspective, it makes sense to ask what new definitions or concepts are supposed to do – what their goal or aim is – and whether they do this well.^28^ Moreover, it makes sense to look at the, perhaps unintended or unforeseen, effects that the introduction and usage of these tools has, and take these into account in the evaluation. As Kingma says: ‘the reason we should care about ideas, concepts and classifications is that they have effects’,^29^ and these effects can be good or bad, desirable or undesirable. Conceptions and classifications of diseases do not exist in a vacuum, but they influence practices, create new realities and have consequences for people’s lives. As such, they are ethically relevant and hence they can, and should, be evaluated from a moral point of view. Taking a pragmatic approach thus implies that a moral evaluation of the effects of a newly proposed concept, definition or classification, becomes an integral part of the evaluation of its defensibility. Whether the reconceptualization of AD is defensible is therefore not merely an epistemic, scientific question, but also a normative, moral issue. So, the questions become: what does the reconceptualization of AD aim to achieve and does it do so well? And what are the practical effects and consequences of this reconceptualization and are they desirable?

## 5 EVALUATING THE CONCEPTS OF PRECLINICAL AND PRODROMAL ALZHEIMER’S DISEASE

Evaluating the reconceptualization of AD in relation to scientific evidence is primarily a task for scientists, and one that is presently being taken on in the Alzheimer’s research literature. We have touched upon that discussion in Section The case, where we pointed out that the amyloid cascade hypothesis is not unanimously accepted among medical scientists, and different conceptualizations of AD, with a less prominent role for amyloid, exist as well.^30^ Here, however, we will focus on evaluating the *aims, goals and effects* – both intended and unintended – of the concepts of preclinical and prodromal AD, referring to a state in which there is no dementia, but abnormal biomarkers presumably related to AD are present.

### 5.1 Aims and goals of the reconceptualization

The first evaluative question to be asked from a pragmatic perspective is: what are the aims of the new concepts and are these attained? From the articles published by the NIA‐AA and IWG it is apparent that the first aim of introducing the new concepts and classification is to enable and support research in this area. In order to perform research into the pathophysiological processes assumed to underlie AD, to test the amyloid hypothesis and to assess the predictive value of various biomarkers, it is necessary to use a common vocabulary and classification system. Likewise, in order to identify suitable research participants for prevention and early intervention trials, and to establish end‐points within such trials and compare trial results, a common lexicon is needed. In this respect, the newly proposed definitions of preclinical and prodromal AD (and their further subclassifications) are partly successful. Although there is no complete consensus, the aim of introducing a new vocabulary to support and align research in the Alzheimer field has been met to a certain extent, considering the establishment of large research programmes with accompanying funding.^31^ The ultimate *goal *is of course to enable early detection and intervention in order to prevent or modify the disease, and hence to decrease the number of people suffering from Alzheimer’s dementia. Whether this goal will be successfully attained, the future will tell. For the moment, no interventions aiming to prevent or modify clinical symptoms of dementia have been successful.

### 5.2 Effects of the reconceptualization on individuals

The next important evaluative question regards the *effects* of the reconceptualization. These can be both intended or unintended – but sometimes foreseeable – and can manifest at either the individual or societal level.

A first concern here is what happens when the terminology that was introduced in the context of research, and intended primarily for communication between researchers, also gets employed in the clinic and in communication with research participants, patients and their family members, as is often the case. The emotional and social effects of terms chosen to communicate with lay‐people can be considerable; being told one is ‘at risk’ for developing AD is different from being told one *has* preclinical or asymptomatic AD – although the situations these terms aim to describe may be exactly the same. Likewise, being told one is in the early stages of AD is different from being told that biomarkers have been detected that may indicate that one is at increased risk of developing dementia within 10–15 years. In dealing with research participants and patients, terminology has different connotations and effects than in communication among researchers. Terms chosen should convey a truthful image of the condition of the person, and not cause confusion, unnecessary anxiety or misconceptions.^32^ Giving someone a diagnosis can be understood as a ‘speech act’: it turns a healthy person or research participant into a patient, which has considerable psychological and social consequences and may be harmful, particularly in the case discussed here, where the person it concerns may never develop any symptoms.^33^

A second concern is that defining these new conditions of ‘being at risk’ or having preclinical and prodromal AD – whatever the exact terms that one uses to describe them – is not necessarily in the best interest of the people involved. As Kutschenko states: ‘diagnostic strategies of clinical research, which aim to obtain well‐defined study populations (e.g., invasive screening techniques) may be scientifically useful but not necessarily serve the needs of clinical practice.’^34^

Apart from the burdens of invasive techniques such as lumbar puncture, the condition these new terms describe may lead to a state of uncertainty and fear for the future. Knowing one is ‘at risk for’ or ‘in the early stages of’ AD can do psychological harm – by creating stress, anxiety, or depression – especially because there is nothing one can do to prevent further development of the disease, i.e., the acquired knowledge is not actionable. However, given the current uncertainty regarding the predictive value of biomarkers and given that a number of people with preclinical or prodromal AD, or MCI due to AD, may never develop dementia, much of this psychological stress may actually be unnecessary. At this moment, there is insufficient evidence regarding the likelihood of such adverse psychological reactions, and it appears that persons who are voluntarily enrolled in clinical trials do understand that biomarkers confer an uncertain risk and may actively seek out that information.^35^ However, the interest in knowing one’s biomarkers decreases when people understand the uncertainty and limited actionability of the information.^36^

Another worry is that people may come to have a different perception of themselves once they know about their condition, start to worry more over normal memory lapses or even develop a nocebo reaction, i.e., show cognitive decline as a result of getting labelled.^37^ Friends and family may also change their attitude or behaviour towards those persons and this may negatively interfere with their relationships.

On the other hand, it has been argued that people do have a right to know their risks, if they want, and that this would enable one to plan and prepare for the future – financially, mentally, with regard to life plans or lifestyle. Even though a risk prediction is not ‘actionable’ in the sense of available preventive or treatment options, it is often claimed it can still be used for such non‐medical purposes.^38^ However, as we have argued elsewhere in more detail, as long as the prognostic value of the diagnosis ‘preclinical AD’ is so unclear and hence risk prediction is inaccurate, this argument has a limited validity.^39^

At present, there is insufficient knowledge about the actual frequency of these potential harms and burdens and about their severity. Little is known about the psychological impact of learning one’s risk status, or learning that one has ‘preclinical’ or ‘prodromal’ AD.

### 5.3 Societal effects

Finally, we should consider the effects of the reconceptualization on a societal level.^40^ Depending on the context and the level of public awareness, a large proportion of the elderly population may eventually prove to be ‘at risk for AD’ or even to have (an early stage of) AD. Although population‐based screening programmes for those over 65 do not seem very likely at this moment, other levels of screening for cognitive impairment may become reality, such as in the context of comprehensive geriatric assessments, which are increasingly popular, or opportunistic screening in those who are presumed to be at risk for cognitive impairment due to clinical or demographic characteristics.

Labelling large numbers of people as such will inevitably raise questions about medicalization of aging and age‐related memory problems, since a growing number of people will be diagnosed with AD without clinical dementia or MCI being present; and without being sure whether they would ever develop it. The number of ‘patients‐in‐waiting’, as they have been called,^41^ would increase enormously. This might also reinforce prejudice about elderly people, stigmatize them and increase age discrimination.

If medication becomes available in the longer run, depending on its effectiveness and risk‐ and side‐effect profiles, this would of course pose new questions. Who should use this medication and how could we prevent over‐medication? How to make sure interests of patients and not only those of the pharmaceutical industry are prioritized? Who should pay for medication, how can equal access be assured, and how much priority should AD prevention get? Would it, at some point, be a good idea to initiate screening programmes and, if yes, in which population?^42^

### 5.4 Taking stock

So, from an individual as well as societal perspective, it is questionable whether the reconceptualization of AD is desirable. Is it a good idea to distinguish, to name and to detect a condition of ‘being at risk for developing AD dementia’ in the first place? The answer is that it might be, in the long run, if the hypothesis on which current research efforts are based proves to be right, and if effective, safe and affordable preventive medication is developed. However, this is by no means a certainty.

For the short term, with the current state of affairs, the answer is likely to be ‘no’. Detecting and labelling an uncertain condition of being at risk – although we do not exactly know how big a risk – for developing AD dementia somewhere in the future does little benefit, as long as the predictive value is unclear and there are no effective preventive measures available.

The reconceptualization is taking out a mortgage on the future success of a specific research agenda, but it may do so at the expense of current research participants, patients and older people in general. This is not necessarily unethical but we should at least be aware of it, weigh the pros and cons and minimize the negative effects. One way to do this is to be very careful with the vocabulary used to address research participants. As Alzheimer Europe states in a recent report: ‘Careful attention should be paid by researchers to the terminology surrounding what is currently defined as pre‐clinical AD and to its possible impact on research participants and the general public.’^43^ They recommend, for example, that research participants falling in the preclinical AD category, should be described as ‘being at risk of AD’ rather than as ‘having preclinical AD’, and that researchers should speak of ‘disclosure of risk status’, rather than of ‘diagnosis’.^44^

Moreover, we believe clinicians who are also involved in research should be acutely aware of these issues and should take utmost care not to conflate research language with clinical language when speaking to patients. We should consider the reconceptualization and the proposed terminology and classification as research tools, not as referring to clinical entities. The AD research community should take responsibility to prevent terms like ‘preclinical AD’, ‘asymptomatic AD’ or ‘at risk for AD’ from prematurely entering the consulting room and the public domain.^45^

## 6 CONCLUSION

While the reconceptualization of AD, and especially the introduction of the notion of preclinical or asymptomatic AD, might seem attractive for research into preventive strategies, and may have the potential to benefit future patients, it will not benefit individuals in the short term. It may lead to diagnosing a pre‐symptomatic condition that in a considerable proportion of cases will never become symptomatic. This can be harmful for individuals and their caregivers. It is important to consider the possible harmful effects of creating these new, uncertain and unclear conditions of pre‐dementia AD in evaluating the defensibility of the proposed reconceptualization. A fundamental shortcoming in the current scientific AD debate is that *illness *is overlooked and that the *disease* is being oversimplified by characterizing it as the presence of biological abnormalities which in themselves correlate poorly with the clinical symptoms of cognitive impairment.

We conclude that the reconceptualization of AD is legitimate and meaningful for usage within a narrowly defined research community studying a clearly defined biological condition, namely the presence of specific measurable biomarkers, but that translation to clinical practice poses various ethical and communication problems. It is too early to move those concepts from research into the clinical setting, since they are based on a disputed hypothesis and since attempts to do so may actually be harmful to the people concerned. The distinction between research and clinical practice may be difficult to maintain, however, and it appears as if the use of biomarkers is slowly creeping into clinical practice, without proper scientific underpinning.^46^

Whether it is a good idea to move toward ever‐earlier diagnosis of AD, or of detecting at‐risk states for AD dementia, is a complex question. A good predictive value and actionability are important preconditions for the ethical implementation of predictive testing. With regard to the first condition, biomarker tests for AD currently fall short, while, for the time being, the second is limited to ‘preparing for one’s future’. Only if the promise of preventive medication materializes, will the reconceptualization of AD turn out to be unequivocally for the better. However, if the attempts to develop such medication continue to fail, the reconceptualization may do more harm than good.

## CONFLICT OF INTEREST

The authors declare no conflict of interest.

## Footnotes

1 Scheltens, P., Blennow, K., Breteler, M. M., de Strooper, B., Fisoni, G. B., Salloway, S., & Van der Flier, W. M. (2016). Alzheimer’s disease. *Lancet*, *388*, 505–517.

2 Ibid.

3 Sperling, R. A., Aisen, P. S., Beckett, L. A., Bennett, D. A., Craft, S., Fagan, A. M., … Phelps, C. H. (2011). Toward defining the preclinical stages of Alzheimer’s disease: recommendations from the National Institute on Aging–Alzheimer’s Association workgroups on diagnostic guidelines for Alzheimer’s disease. *Alzheimer’s Dementia*, *7*(3), 280–292; Dubois, B., Feldman, H. H., Jacova, C., Cummings, J. L., Dekosky, S. T., Barberger‐Gateau, P., … Scheltens, P. (2010). Revising the definition of Alzheimer’s disease: A new lexicon. *Lancet Neurology*, *9*, 1118–1127; Dubois, B., Feldman, H. H., Jacova, C., Hampel, H., Molinuevo, J. L., Blennow, K., … Cummings, J. L. (2014). Advancing research diagnostic criteria for Alzheimer’s disease: The IWG‐2 criteria. *Lancet Neurology*, *13*, 614–629.

4 Fox, P. (1989). From senility to Alzheimer's disease: The rise of the Alzheimer’s disease movement. *The Milbank Quarterly*, *67*(1), 58–102

5 Whitehouse, P. J. (2007). Mild cognitive impairment – A confused concept? *Nature Clinical Practice.* *Neurology*, *3*(2), 62–63; Petersen, R. C. (2007). Mild cognitive impairment: current research and clinical implications. *Seminars in Neurology*, *27*(1), 22–31.

6 Dubois et al. (2014), *op. cit.* note 3; Sperling et al., *op cit.* note 3.

7 Jack, C. R., Knopman, D. S., Jagust, W. J., Shaw, L. M., Aisen, P. S., Weiner, M. W., … Trojanowski, J. Q. (2010). Hypothetical model of dynamic biomarkers of the Alzheimer’s pathological cascade. *Lancet Neurology*, *9*(1), 119–128.

8 Schneider, J. A., Arvanitakis, Z., Bang, W., & Bennett, D. A. (2007). Mixed brain pathologies account for most dementia cases in community‐dwelling older persons. *Neurology*, *69*, 24; Savva, G. M., Wharton, S. B., Ince, P. G., Forster, G., Matthews, F. E., Brayne, C.; Medical Research Council Cognitive Function and Ageing Study. (2009). Age, neuropathology, and dementia. *New England Journal of Medicine*, *360*, 2302–2309.

9 Of course one could argue that those with Alzheimer changes without dementia simply died before they had the chance to develop symptoms and those with a clinical diagnosis of Alzheimer’s dementia who did not have AD pathology were misdiagnosed during life. Still, these data, as well as the data on MCI, cast doubt on the validity of the amyloid cascade hypothesis.

10 Heister, D., Brewer, J. B., Magda, S., Blennow, K., McEvoy, L. K.; Alzheimer’s Disease Neuroimaging Initiative. (2011). Predicting MCI outcome with clinically available MRI and CSF biomarkers. *Neurology*, *77*, 1619–1628.

11 Wolfsgruber, S., Polcher, A., Koppara, A., Kleineidam, L., Frölich, L., Peters, O., … Wagner, M. (2017). Cerebrospinal fluid biomarkers and clinical progression in patients with subjective cognitive decline and mild cognitive impairment. *Journal of Alzheimer’s Disease*, *58*, 939–950.

12 Boorse, C. (1977). Health as a theoretical concept. *Philosophy of Science*, *44*(4), 542–573; Boorse, C. (1997). A rebuttal on health. In J. M. Humber & R. F. Almeder (Eds*.*), *What* *is disease? *(pp. 3–133). Totowa, NJ: Humana Press; Nordenfelt, L. (1995). *On* *the nature of health.* Dordrecht, Netherlands: Kluwer Academic Publishers; Nordenfelt, L. (2007). The concepts of health and illness revisited. *Medicine, Healthcare and Philosophy*, *10*, 5–10.

13 Boorse 1977, *op cit*. note 12; Boorse 1997, *op cit*. note 12.

14 As mentioned above, there is some doubt about the accuracy of the amyloid cascade hypothesis, and the question of whether biomarkers do indeed represent pathology, i.e., abnormal physiological functioning.

15 Savva et al., *op. cit.* note 8; Richard, E., Schmand, B., Eikelenboom, P., Westendorp, R. G., & Van Gool, W. A. (2012). The Alzheimer myth and biomarkers research in dementia. *Journal of Alzheimer’s Disease*, *31*(s3), S203–S209.

16 In a recent paper, however, Boorse seems open to the idea that young adults should be taken as the reference group for *all *adults, which would imply that various aspects of ‘normal aging’ should be considered pathological in many cases. See: Boorse, C. (2014). A second rebuttal on health. *Journal of Medicine and Philosophy*, *39*(6), 683–724.

17 Nordenfelt 1995, *op. cit*. note 12; Nordenfelt 2007, *op. cit*. note 12.

18 Giaccone, G., Arzberger, T., Alafuzoff, I., Al‐Sarraj, S., Budka, H., Duyckaerts, C., … Winblad, B. (2011). New lexicon and criteria for the diagnosis of Alzheimer’s disease. *Lancet Neurology*, *10*, 298–299.

19 Hofmann, B. (2002). On the triad disease, illness and sickness. *Journal of Medicine and Philosophy*, *27*(6), 651–673.

20 Kingma, E. (2013). Health and disease: social constructivism as a combination of naturalism and normativism. In H. Carel & R. Cooper (Eds.), *Health, illness & disease.* *Philosophical essays* (pp. 37–56). Durham, UK: Acumen.

21 George, D. R., Qualls, S. H., Camp, C. J., & Whitehouse, P. J. (2013). Renovating Alzheimer’s: ‘Constructive’ reflections on the new clinical and research diagnostic guidelines. *The Gerontologist*, *53*(3), 378–387.

22 This does not imply that diseases are *merely* constructions, or that they are arbitrary. It just claims that the natural world does not *by itself* determine the way in which we ought to ‘carve her up’; we make decisions about that.

23 Hacking, I. (1999). *The social construction of what?* Cambridge, MA: Harvard University Press; Zachar, P. (2002). The practical kinds model as a pragmatist theory of classification. *Philosophy, Psychiatry & Psychology*, *9*(3), 219–227.

24 Schneider, L. S. (2010). Organising the language of Alzheimer’s disease in light of biomarkers. *Lancet Neurology*, *9*, 1045.

25 Jack et al., *op. cit.* note 7.

26 George et al., *op. cit.* note 21: ‘Many of the authors of the guidelines are consultants to drug companies, and the field itself is strongly influenced by the pharmaceutical industry, whose economic interests powerfully shape and influence human comprehension of biological processes.’

27 See for example, Zachar, *op. cit.* note 23; Agich, G. (1997). Toward a pragmatic theory of disease. In J. M. Humber & R. F. Almeder (Eds.),* What* *is disease?* (pp. 219–246). Totowa, NJ: Humana Press; Ross, P. (2005). Sorting out the concept of disorder. *Theoretical Medicine and Bioethics*, *26*, 115–140. Severinsen, M. (2001). Principles behind definitions of diseases – A criticism of the principle of disease mechanism and the development of a pragmatic alternative. *Theoretical Medicine*,* 22*, 319–336.

28 Petersen, R. C. (2013). Do preclinical Alzheimer’s disease criteria work? *Lancet Neurology*, *12*, 935.

29 Kingma, *op. cit.* note 20, p. 45.

30 Richards and Brayne state: ‘There exists a very deep disagreement about what Alzheimer’s disease is. According to the amyloid hypothesis, it is a clinico‐pathological entity consisting of specific brain pathology (plaques and tangles) and a specific set of clinical symptoms. According to other experts, Alzheimer’s disease is a “diffuse clinical syndrome representing the gradual accumulation of multiple pathologies, arising from multiple interlocking risk factors over the life course”.’ Richards, M., & Brayne, C. (2010). What do we mean by Alzheimer’s disease? *British Medical Journal*, *341*, 865–867.

31 Ritchie, C. W., Molinuevo, J. L., Truyen, L., Satlin, A., Van der Geyten, S., Lovestone, S.; European Prevention of Alzheimer’s Dementia (EPAD) Consortium. (2016). Development of interventions for the secondary prevention of Alzheimer’s dementia: the European Prevention of Alzheimer’s Dementia (EPAD) project. *Lancet Psychiatry*, *3*, 179–186.

32 Kutschenko, L. K. (2012). Diagnostic misconceptions? A closer look at clinical research on Alzheimer’s disease. *Journal of Medical Ethics*, *38*(1), 57–59.

33 Ibid., 58.

34 Ibid.

35 Bemelmans, S. A. S. A., Tromp, K., Bunnik, E. M., Milne, R. J., Badger, S., Brayne, C., … Richard, E. (2016). Psychological, behavioral and social effects of disclosing Alzheimer’s disease biomarkers to research participants: a systematic review. *Alzheimer’s Research & Therapy*, *8*, 46; Mozersky, J., Sankar, P., Harkins, K., Hachey, S., & Karlawish, J. (2018). Comprehension of an elevated amyloid positron emission tomography biomarker result by cognitively normal older adults. *JAMA Neurology*, *75*, 44–50.

36 Milne, R., Diaz, A., Richard, E., Badger, S., Gove, D., Georges, J., … Brayne, C. (2018). Perspectives on communicating biomarker‐based assessments of Alzheimer’s disease to cognitively healthy individuals. *Journal of Alzheimer’s Disease*, *62*, 487–498.

37 Lineweaver, T. T., Bondi, M. W., Galasko, D., & Salmon, D. P. (2014). Effect of knowledge of APOE genotype on subjective and objective memory performance in healthy older adults. *American Journal of Psychiatry*, *171*, 201–208.

38 For example, Vanderschaeghe, G., Schaeverbeke, J., Vandenberghe, R., & Dierickx, K. (2017). Amnestic MCI patients’ perspectives toward disclosure of amyloid PET results in a research context. *Neuroethics*, *10*, 281–297; Holt, G. R. (2011). Timely diagnosis and disclosure of Alzheimer disease gives patients opportunities to make choices. *Southern Medical Journal*, *104*, 779–780.

39 Bunnik, E. M., Richard, E., Milne, R., & Schermer, M. H. N. (2018). On the personal utility of Alzheimer's disease‐related biomarker testing in the research context. *Journal of Medical Ethics*. https://doi.org/10.1136/medethics-2018-104772

40 Karlawish, J. (2011). Addressing the ethical, policy, and social challenges of preclinical Alzheimer disease. *Neurology*, *77*, 1487–1493; Boenink, M., Cuijpers, Y., van der Laan, A. L., van Lente, H., & Moors, E. (2011). Assessing the sociocultural impacts of emerging molecular technologies for the early diagnosis of Alzheimer's disease. *International Journal of Alzheimer’s Disease*, Article ID 184298; Schicktanz, S., Schweda, M., Ballenger, J. F., Fox, P. J., Halpern, J., Kramer, J. H., … Jagust, W. J. (2014). Before it is too late: professional responsibilities in late‐onset Alzheimer's research and pre‐symptomatic prediction. *Frontiers in Human Neuroscience*, *20*, 921.

41 Timmermans, S., & Buchbinder, M. (2010). Patients‐in‐waiting: Living between sickness and health in the genomics era. *Journal of Health and Social Behavior*, *51*, 408.

42 Le Couteur, D. G., Doust, J., Creasey, H., & Brayne, C. (2013). Political drive to screen for pre‐dementia: not evidence based and ignores the harms of diagnosis. *British Medical Journal*, *347*(9), f5125.

43 Alzheimer Europe. (2016). Discussion paper on ethical issues linked to the changing definitions/use of terms related to Alzheimer’s disease. Retrieved from https://www.alzheimer-europe.org/Publications/Alzheimer-Europe-Reports

44 Ibid.

45 The IWG actually agrees with this: ‘because of the uncertainty [about how big the risk for developing AD dementia is] and for ethical reasons, we emphasise that reference to preclinical AD should be avoided. At the diagnostic or clinical level, any (clinically asymptomatic) individual should be described as being “at risk for AD” … but should not be defined as having preclinical AD.’ Dubois et al. (2010), *op. cit.* note 3, p. 1122.

46 A telling sign is the way in which some publications simply replace the term ‘research criteria’ with ‘clinical criteria’, thereby completely bypassing the proclaimed intentions of the working groups as well as the differences between research and clinical practice. See for example: Morris, J. C., Blennow, K., Froelich, L., Nordberg, A., Soininen, H., Waldemar, G., … Dubois, B. (2014). Harmonized diagnostic criteria for Alzheimer’s disease: Recommendations. *Journal of Internal Medicine*, *275*, 204–213. In another consensus paper, several references to the adoption in clinical practice are made, such as ‘will be available in the near future for people diagnosed with prodromal dementia’ and ‘healthcare systems will need to identify and engage with prodromal and preclinical populations’. Ritchie C. W., Russ, T. C., Banerjee, S., Barber, B., Boaden, A., Fox, N. C., … Burns, A. (2017). The Edinburgh Consensus: Preparing for the advent of disease‐modifying therapies for Alzheimer’s disease. *Alzheimer’s Research and Therapy*, *1*, 85.

